# Understanding levels of best practice: An empirical validation

**DOI:** 10.1371/journal.pone.0198888

**Published:** 2018-06-14

**Authors:** Huy P. Phan, Bing H. Ngu, Hui-Wen Wang, Jen-Hwa Shih, Sheng-Ying Shi, Ruey-Yih Lin

**Affiliations:** 1 School of Education, University of New England, Armidale, NSW, Australia; 2 Department of Philosophy, Huafan University, New Taipei City, Taiwan; 3 Department of Buddhist Studies, Huafan University, New Taipei City, Taiwan; 4 Graduate Institute of Asian Humanities, Huafan University, New Taipei City, Taiwan; 5 Industrial Engineering and Management Information, Huafan University, New Taipei City, Taiwan; Indiana University, UNITED STATES

## Abstract

Recent research has explored the nature of the theoretical concept of *optimal best practice*, which emphasizes the importance of personal resolve, inner strength, and the maximization of a person’s development, whether it is mental, cognitive, social, or physical. In the context of academia, the study of optimal functioning places emphasis on a student’s effort expenditure, positive outlook, and determination to strive for educational success and enriched subjective well-being. One major inquiry closely associated with optimal functioning is the *process of optimization*. Optimization, in brief, delves into the *enactment of different psychological variables* that could improve a person’s internal state of functioning (e.g., cognitive functioning). From a social sciences point of view, very little empirical evidence exists to affirm and explain a person’s achievement of optimal best practice. Over the past five years, we have made extensive progress in the area of optimal best practice by developing different quantitative measures to assess and evaluate the importance of this theoretical concept. The present study, which we collaborated with colleagues in Taiwan, involved the use of structural equation modeling (SEM) to analyze a cohort of Taiwanese university students’ (*N* = 1010) responses to a series of Likert-scale measures that focused on three major entities: (i) the importance of optimal best practice, (ii) three major psychological variables (i.e., effective functioning, personal resolve, and emotional functioning) that could optimize student’ optimal best levels in academic learning, and (iii) three comparable educational outcomes (i.e., motivation towards academic learning, interest in academic learning, and academic liking experience) that could positively associate with optimal best practice and the three mentioned psychological variables. Findings that we obtained, overall, fully supported our initial *a priori* model. This evidence, in its totality, has made substantive practical, theoretical, and methodological contributions. Foremost, from our point of view, is clarity into the psychological process of optimal best practice in the context of schooling. For example, in relation to subjective well-being experiences, how can educators optimize students’ positive emotions? More importantly, aside from practical relevance, our affirmed research inquiry has produced insightful information for further advancement. One distinction, in this case, entails consideration of a more complex methodological design that could measure, assess, and evaluate the impact of optimization.

## Introduction

*Optimal best practice* in educational contexts is concerned with an individual’s maximization of his/her academic capability. A student’s self-awareness of his/her optimal best practice may yield reflective consideration, such as: “What is the best that I can do for this subject?”, “I can achieve a score of 90/100”, and “This is the best that I do”. This theoretical concept, focusing on a state of exceptionality in the learning process, reflects the *paradigm of positive psychology* [1–3], which recognizes the importance of resilience, inner strengths and virtues, an internal state of flourishing, and proactivity in human functioning (e.g., a heightened state of emotional functioning).

Researchers and educators, to date, have focused on different inquiries into the study of optimal best practice. Motivational theorists have inquired into the explanatory effects of different motivational concepts, for example: *achievement goals* [4, 5], and *expectations-values of learning tasks* [6, 7]. Other researchers have specifically explored and addressed the operational definitions and characteristics of optimal best [8–10]. This line of research, which is still in its early stage of evolution, is concerned with the *operational nature of optimal best*–that is, how do we measure and assess the concept of optimal best?, how does an individual experience and achieve optimal best in a subject matter?, and what impact does optimal best have on different adaptive outcomes?

**Fig 1:**
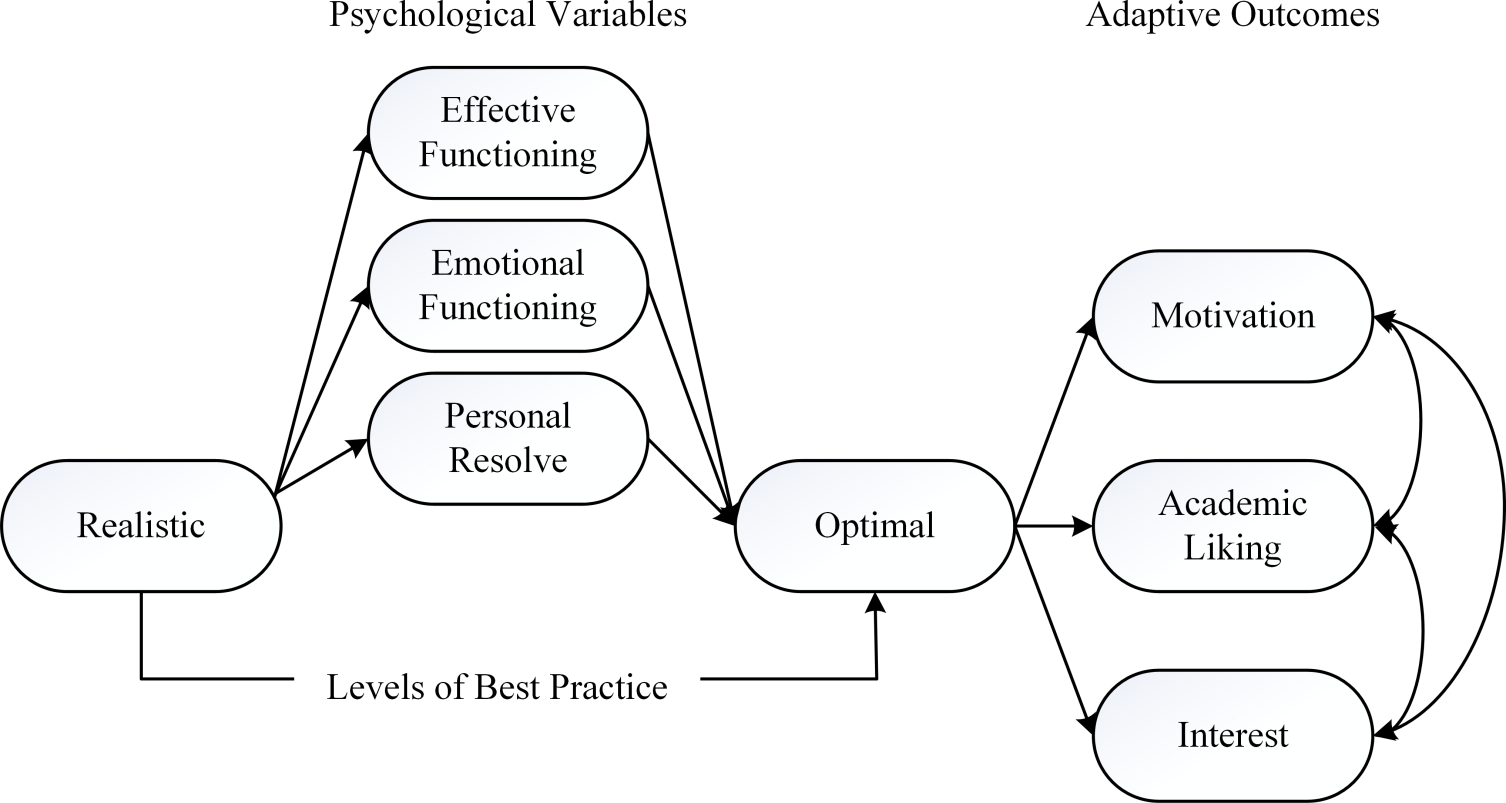
Conceptual model.

This article describes our recent undertaking, which involved the testing of a conceptualized structural model that focused on the operational nature of optimal best. We draw on recent research progress [8–12] to develop a conceptualization that is noteworthy for investigation. Our major inquiry, as shown in Fig 1, hypothesizes the potential intricate associate between a person’s current knowledge base and his/her experience of optimal best. At the same time, expanding previous theorizations [10, 13], we postulate the positive influences of three major psychological variables on optimal best: *effective functioning*, *emotional functioning*, and *personal resolve*. Moreover, as a distinctive entity, we argue that experience of optimal best would result in the prediction of two interrelated adaptive outcomes: *motivation towards learning* and *interest in learning tasks*.

## In brief: The importance of optimal best practice

The study of *optimal best* is innovative for its inquiries into individuals’ optimal experiences in different subject matters. Csíkszentmihályi [3], for example, has made reference to the concept of *cognitive flow*, which connotes an individual’s state of optimal experience. The paradigm of positive psychology, reflecting the work of Seligman and others [1, 2, 14], recognize optimal experience in terms of a person’s *inner strengths*, *virtues*, *resilience*, and *proactivity*. Fraillon’s [10] theoretical of the topical theme of subjective well-beings emphasizes the importance of *optimization*. Diener and his colleagues [15, 16], likewise, introduced the concept of *flourishing* and equating it as a component of a person’s subjective well-being. Flourishing in its simplistic term, according to Huppert and So [17], refers to a person’s experience of life that is going well. Another theoretical concept related to subjective well-being is that of *thriving*, defined by [18] as a person’s state of positive functioning at its fullest range–mentally, physically, and socially (p. 254).

In the area of student motivation, Martin and his colleagues [8, 12, 13, 19] have explored the concept of *personal best goals*, which delves into the notion of personalized standards of excellence. Students accomplish personal best goals when the performance that they attain and the effort that they expend are higher than, or is good as, their previous best performance and effort [8]. Personal best goals, according to the authors, are significant in terms of their predictive influences on different types of educational outcomes (e.g., deep learning).

In a similar vein, capitalizing on Fraillon’s [10] description of the process of optimization, Phan and his colleagues [9, 20] recently provided a theoretical description of the concepts of *best practice*, which entails two distinctive levels: (i) *realistic best practice* (i.e., a person’s actual competence, at present) and (ii) *optimal best practice* (i.e., the maximization of a person’s competence, at present). According to the authors’ theorization, achieving personal experience of optimal best practice from realistic best practice (i.e., RL–OL) requires the facilitation of different psychological processes, such as personal self-efficacy [21]. Unlike previous conceptualizations (e.g., flourishing [15], personal best goals [12]), however, empirical research development into the RL–OL difference has received moderate attention, to date.

Within the context of academia, other conceptualizations that have been studied to determine students’ motivational beliefs, positive school experiences, and academic learning outcomes. Aside from the importance of *personal self-beliefs* [21], researchers have also focused on the potent effects of *academic buoyancy* [22–24], *optimism* [25, 26], *hope* [27, 28], and *academic striving* [29, 30]. These psychological constructs, in terms of their similarities, serve as potent determinants of different types of adaptive outcomes.

From the preceding sections then, taking into account different conceptualizations and research inquiries [13, 15, 18], optimal best epitomises the *maximization of a person’s capability to be successful in a course of action*. Optimal best is positive in terms of its characteristics and contrasts to that of stagnation, pessimism, and a low state of functioning–cognitively, emotionally, and/or physically. In the context of academia, personal experience of optimal best may indicate a student’s seeking of mastery in a subject matter for inner satisfaction and/or personal improvement, or his/her exceptional result in a performance test. A student’s indication of optimal best, in this sense, may reflect his/her state of personal resolve, resilience, and motivation for learning.

## The present study: Validating the concept of optimal best

Understanding an individual’s optimal best practice in academic contexts is an important focus of inquiry for development. This emphasis is poignant and enables educators to encourage academic exceptionality, regardless of individual variations–for example, what is the best that you can do?, do you know your personal best?, and how can we assist you to achieve your optimal best? The present study is innovative for its research expansion into existing conceptualizations of optimal best practice [9, 10, 12]. Optimal best practice is considered as *an achievement of exceptionality*, reflecting the maximization of a person’s capability in a subject matter, and/or a situational circumstance.

Unlike previous research inquiries into the concept of optimal best [12, 13, 18], we postulate that a ‘point of reference’ for personal comparison is needed. Fraillon [10], in particular, has coined this point of reference as ‘actual best functioning’ whereas Phan et al. [9] have theoretically termed it as ‘realistic best practice’. Realistic best practice or actual best functioning, as we have discussed, indicate what a person is capable of, at present (e.g., I can solve this Algebra problem (e.g., *x* + 10 = -9), or I am very happy at the moment). Capitalizing on this tenet, we argue that a person’s actual best functioning could form the basis for the striving and development of his/her optimal best, which Fraillon [10] refers to it as ‘notional best functioning’. In other words, from our proposition, understanding of the nature and characteristics of optimal best requires an *observable index of current practice*, which a person would benchmark against. Moreover, from our point of view, taking into account existing theorizations [21, 31, 32], we contend that there are personal and extraneous influences that define and shape a person’s current level of best practice. For example, in accordance with Bandura’s [21] *social cognitive theory*, we acknowledge that a person’s enactive learning experiences (e.g., repeated successes in a subject matter), subject to both mastery and performance-based criteria, could determine his/her current level of best practice–a secondary school student who has repeatedly failed in different topics of mathematics, in this case, is more likely to report a low-moderate level of current best practice. According to Bandura [21], and affirmed by a number of researchers [33–35], enactive learning experience is one of the most potent sources of information in the prediction of a person’s motivational beliefs.

Our research inquiry, as shown in Fig 1, addresses the pervasive issue of the *operational nature of optimal best*, which encompasses two major themes for consideration: (i) the extent to which comparable psychological variables could facilitate the achievement of experience of optimal best, and (ii) the impact that experience of optimal best would have on different types of educational outcomes. This research investigation, as we rationalize in the next section of the article, is innovative for its scope into an explanatory account of how a person reaches an optimal level. To our knowledge, from the existing literature, no research has yet considered the use of a reference point (i.e., a person’s current knowledge base) as a basis to investigate the nature of optimal best.

### Psychological variables for consideration

Our conceptualization for examination postulates understanding into the achievement of optimal best practice (OPB) requires a reference point for determination, denoted in this case as realistic best practice (RBP). This consideration, as we have explained, suggests that there is a quantifiable difference between a person’s current knowledge based and his/her optimal best functioning (i.e., RBP–OPB). According to Fraillon [10], progressing from actual best functioning (e.g., knowing how to solve a simple arithmetic problem: 5 + ___ = -10) to that of notional best functioning (e.g., knowing how to solve an arithmetic problem with two unknowns: *x* + *y* = 10 and 2*x* –*y* = 5) is intricately linked to the internal process of optimization, which may involve the operational functioning of *psychological processes* (e.g., the role of hope)[36]), *educational practices* (e.g., an appropriate instructional design)[37]), and/or *psychosocial factors* (e.g., the impact of the home environment)[38]).

Aside from the acquiring of subject content, as shown here, the impact of a psychological variable (PV) may enable the achievement of OBP from RBP. We argue that, in this case, there are three comparable psychological variables that could operate to facilitate the RBP–OBP difference:

#### i. Effective functioning

Fraillon’s [10] theoretical mentioning of the optimization of subjective well-being experiences included the concept of effective functioning, which is concerned with an evaluation of how a person’s responses to his/her contextual environment support his/her functioning to fulfil the complexity of that environment. An elaborated definition of effective functioning, recently revised, focuses on a person’s purposive state of organization, structured thoughts and behavioural patterns, and his/her deliberate intent to succeed in life [39, 40].

Effective functioning, in this sense, reflects the importance of efficiency, which takes into account the existence and availability of resources, a person’s time, and his/her expenditure of effort. This theorization [10, 40] postulates that effective functioning may serve to motivate and elicit positive educational outcomes. A purposive state of organization, structured thinking, and purposive intent may direct a person to persist in the course of his/her academic learning, regardless of obstacles and difficulties. In their longitudinal study involving secondary school students, Phan, Ngu, and Alrashidi [40] found that the concept of effective functioning positively predicted both academic achievement (β = .30, *p* < .001) and the enrichment of school experience (β = .62, *p* < .001). In another research investigation, Phan and Ngu [39] reported similar findings: the positive effect of effective functioning on academic achievement (β = .17, *p* < .01) and personal self-efficacy (β = .62, *p* < .001).

This consistent evidence, albeit preliminary at this stage, empirically supports our conceptualization into the potentiality for effective functioning to optimize and facilitate a person’s achievement of OBP in a subject matter. The characteristics of planning and organization, structured thinking (e.g., what should I do next?) and deliberation, and self-awareness of efficiency (e.g., I need to be mindful of my time and effort), for example, may assist a person to focus on a deliberate course of action in order to progress from RBP to OBP.

#### ii. Emotional functioning

Enriched subjective well-being experiences [10, 41] and the paradigm of positive psychology [1, 2] connote the importance of a person’s state of emotional functioning. Emotional functioning, in fact, is a major component of the totality of a person’s subjective well-being. This theoretical concept delves into a person’s understanding and management of his/her emotions, placing emphasis on positive emotions (e.g., happiness).

Awareness of one’s own positive emotions plays an important role in the enhancement of capacity building for optimistic thinking, problem solving, and decision making and to lead to more flexible, innovative and creative solutions [41–43]. Negative emotions, in contrast, have been noted to deter and undermine students’ academic learning experiences and performance outcomes [44–46]. Pajares’ earlier work into the central role of personal self-efficacy [21], for example, showed the negative effect of a heightened state of anxiety [44, 47].

Similar to the conceptualization of effective functioning, we contend that positive emotional functioning could serve to optimize and facilitate a person’s OBP. Personal experience of positive emotions is more likely, in this case, to instil a sense of confidence and motivation to enable a person to achieve OBP. By the same token, in contrast, we argue that negative emotional functioning could act as an obstacle to deter a person from reaching OBP. A state of anxiety, for example, may instil a sense of helplessness and frustration, which could then result in disengagement and withdrawal from the situation and/or subject matter.

#### iii. Personal resolve

Research into subjective well-beings has also recognized the importance of personal resolve, which is similar to that of a person’s state of resilience [48, 49]. Personal resolve, aligning to the process of optimization [10], is concerned with a person’s internal state of resolve and decisiveness to strive for optimal best practice in an optimistic manner [40]. This theoretical concept emphasizes the importance of determination to overcome any obstacle that may arise, and to engage in a purposive act in order to self-fulfil the internal desire of achieving OBP.

Experience of personal resolve, in accordance with the paradigm of positive psychology [1, 2], demonstrates and reflects a person’s inner strengths and resilience to succeed in life. In contrast, of course, is a lack of personal resolve indicates indecisiveness, weakness, and procrastination to progress in a course of action. Possessing a strong level of personal resolve may direct focus and motivate a person to strive for OBP. In our recent study [39], which involved secondary school students, we found that personal resolve positively influenced academic achievement (β = .16, *p* < .05). Phan et al.’s [40] earlier longitudinal research, likewise, reported the impact of personal resolve on contextualized self-efficacy beliefs (e.g., personal resolve → task-specific self-efficacy for academic learning: β = .14, *p* < .05).

From the above description then, we postulate that experience and achievement of OBP from RBP requires the facilitation of different psychological processes. Achieving OBP from RBP may consist of cognitive maturity, acquiring new content and pedagogical knowledge and, of course, the enactment of psychological variables. Our inquiry in this matter seeks clarity into the first aspect of the operational nature of OBP, namely: the *extent to which comparable psychological variables could facilitate OBP*.

### Different types of adaptive outcomes

Experience of OBP, we contend, is a noteworthy entity for personal development. By its definition [8, 9, 12], optimal best practice in educational contexts reflects an exceptional state of cognitive functioning–for example, repeated academic successes that are outstanding. The potency of OBP in a subject matter, in this instance, may involve a measure of its predictive effect onto individual related outcomes. In relation to secondary school mathematics learning, for example, OBP in this case may indicate an exceptional result in the half-yearly exam or receiving 1^st^ Class Honours Distinction. At the same time, however, we postulate that OBP may analogously align to three comparable outcomes that have been studies by Van Damme and his colleagues: *motivation towards learning* (i.e., a student’s state of motivation towards his/her academic learning), *interest in learning tasks* (i.e., a student’s indication of his/her interest regarding the learning of different subject matters), and *academic liking experience* (i.e., a student’s emotions, feelings, and preference of academic experience)[11, 50–52].

The potential impact of OBP on the three aforementioned achievement-related outcomes is worth investigating. We argue that achievement of OBP would closely correspond with high reported scores for motivation towards learning, interest in learning tasks, and academic liking experience, all of which indicate a proactive state of academic engagement for learning. The inability to achieve OBP, in contrast, would yield comparable low scores, which associate with a state of disengagement and amotivation from the schooling process, in general. This postulation, we contend, emphasizes the saliency of the nature of OBP–that is, its potential explanatory power to predict and improve achievement-related outcomes. As a point of comparison, for example, we recently found that the concept of relating to others (e.g., teacher-student relationship) positively predicted motivation towards learning (β = .17, *p* < .01) and academic liking experience (β = .24, *p* < .001)[53]. In another study that involved the use of path analysis techniques, we reported on the positive effects of global self-esteem (β = .15, *p* < .05) and domain-specific self-esteem (β = .25, *p* < .001) on the concept of interest in learning tasks.

In sum, from the preceding section, we argue that personal experience of OBP is a central feat of human agency. This postulation, situated within the context of academia, focuses on the potentiality for OBP to better a person’s learning experience, performance outcomes, and state of motivation. OBP, in this case, from our conceptualization, does not limit itself to the task or goal (e.g., to achieve 1^st^ Class Honours Distinction in Psychology 101) at hand, but rather expand to encompass other educational outcomes. The second aspect of the operational nature of OBP, in this case, makes attempts to address *whether* and/or *the extent to which OBP would predict motivation towards learning*, *interest in learning tasks*, *and academic liking experience*.

## Conceptualization of the present study

We conceptualize the research inquiry for undertaking based on existing theorizations and empirical evidence. The main focus of the study makes attempts to inquire into the personal experience of optimal best in the schooling process. Optimal best, in this case, is concerned with a person’s exceptional best in a subject matter–for example, a secondary school student’s achievement of a ranking in Literacy may indicate his/her personal best. What is of interest for us then, from a quantitative methodological approach, is an inquiry into the operational nature of OBP–that is, in accordance with our previous discussion, the operational nature of OBP encompasses understanding of *its cause and explanatory power*.

Our methodological approach, correlational in nature, addresses the fundamental associations between the two levels of best practice–namely, the direct impact of RBP on OBP, and the extent to which the three psychological variables could act in ‘between’ the two levels of best practice. We postulate that RBP, as an exogenous variable, could serve as a powerful source of information in the direct facilitation of OBP, as an endogenous variable. This consideration places emphasis on a person’s current knowledge base (i.e., his/her realistic best), which could inform and motivate a person to strive for the *status quo*. In the context of academia, a student may gauge into his/her current knowledge base to make decisions, engage in the learning process and, more importantly, strive for achievement of optimal best.

In a similar vein, from previous research development [8, 10, 12, 40], we argue that appropriate psychological variables could also facilitate and assist in the prediction of OBP. Because of their theorized positive characteristics, we argue that effective functioning, emotional functioning, and personal resolve could directly predict OBP. At the same time, we consider the extent to which RBP could also indirectly influence OBP, via the three mentioned psychological variables. Correlational research studies have, to date, produced evidence that indicates the potent effects of emotional functioning (e.g., happiness)[54]), effective functioning, and personal resolve on different types of adaptive outcomes [39, 40]. Finally, consideration of the potent influence of OBP is made with the inclusion of motivation towards learning, interest in learning tasks, and academic liking experience as possible outcomes.

## Methods

### Sample and procedure

A total sample of 1010 undergraduate students (*N* = 405 males, 605 females) from seven universities (i.e., two public universities, five private universities) located in Taipei City and New Taipei City, Taiwan took part in the study. In Taiwan, there are two types of university: (i) private university, which is private and privately funded by the student, himself/herself, and (ii) public university, which public and more prestigious and competitive, in nature. The majority of the participants, in this case, were from the private universities (*N* = 878). Entry into a public university in Taiwan (e.g., National Taiwan University) is an extremely competitive process, relying on high academic results. Students who do not meet the cut-off threshold into a public university proceed then onto entry into private universities.

The participants voluntarily took part in the study, knowing that there were no incentives and that they could withdraw from the study anytime during the course of the data collection process. The questionnaires were administered using a paper-format in lectures and tutorial classes. The questionnaires took approximately 25–30 minutes to complete, and participants were encouraged to ask for clarification at the end, if necessary. The questionnaires consisted of a front-page demographic information sheet, which required the participants to indicate the following: gender (e.g., male), university (e.g., National Taiwan University), department (e.g., Department of Engineering), course of study (e.g., Bachelor of Liberal Arts), age, and study status (e.g., Full-time).

The medium of formal instruction at school and in university is Chinese Mandarin. The questionnaires, originally conceptualized in English, were translated to Chinese Mandarin for the participants. A three-step methodological procedure was undertaken: (i) the questionnaires were first translated from English to Chinese Mandarin by one of the authors and another Ph.D. student at one of the Taiwanese universities (Note: the Ph.D. student also specialized in the study of the subject ‘English as a Foreign Language’), (ii) the questionnaires, now in Chinese Mandarin, were back-translated to English by a staff at one of the Taiwanese universities (Note: the staff teaches ‘English as a Foreign Language’) and another author of this article, who is also a native speaker of both English and Chinese Mandarin, and (iii) cross-checking was made with the English-Chinese Mandarin translation and the Chinese Mandarin-English translation, in total, to ensure consistency and accuracy with the original scales.

### Instruments

We used existing Likert-scale inventories to measure and assess the mentioned concepts. For consistency, we structured the subscales to consist of five ratings: 1 (Completely Disagree) to 5 (Completely Agree). Furthermore, in this section, we report on the results of the psychometric properties of the six scales. We used confirmatory factor analysis (CFA) techniques [55, 56] to explore the factorial structure of each scale. Specifically, we performed a one-factor congeneric model to determine the appropriateness of the factor loadings of items of each scale. To determine the goodness-of-fit of each congeneric model, we used the threshold values of the following goodness-of-fit indexes: the χ^2^/d*f* ratio, the Comparative Fit Index (CFI)(i.e., CFI value > .95), the Tucker Lewis Index (TLI)(i.e., TLI value > .95), the Root Mean Square Error of Approximation (RMSEA)(i.e., RMSEA value < .07), and the Standardized Root Mean Square Residual (SRMR)(i.e., SRMR value < .05).

#### Realistic best practice

We adapted from the Optimal Outcome Questionnaire [57] and developed five items to measure and assess the concept of RBP [20]. The five items included, for example: “I am content with what I have accomplished so far at this university” and “I can achieve what is being asked of me at this university”. A one-factor congeneric model analysis of this model, Model M_1_, showed a moderate fit, as indicated by the following: χ^2^/d*f* = 12.31, *p* < .001, CFI = .94, TLI = .87, RMSEA = .11 (Lo90 = .08, Hi90 = .13), *p* < .001, and SRMR = .04. We respecified this a priori model with the inclusion of an error variance between Item 4 and Item 5. The goodness-of-fit index values for this a posteriori model, Model M_2_, showed an improvement in model fit: χ^2^/d*f* = 8.68, *p* < .001, CFI = .96, TLI = .91, RMSEA = .09 (Lo90 = .06, Hi90 = .12), *p* < .01, and SRMR = .03. The Δχ^2^ test between the two models was statistically significant, *p* < .001 (i.e., Δχ^2^_(Model M1 –Model M2)_ = 26.81), indicating support for the a posteriori model. To improve the fit further, we respecified Model M_2_ with the inclusion of an error variance between Item 3 and Item 4. The goodness-of-fit index values for this modified model, Model M_3_, improved over that of Model M_2_: χ^2^/d*f* = 7.46, *p* < .001, CFI = .98, TLI = .94, RMSEA = .07 (Lo90 = .05, Hi90 = .09), *p* < .05, and SRMR = .02. The Δχ^2^ test between the two models was statistically significant, *p* < .001 (i.e., Δχ^2^_(Model M1 –Model M2)_ = 12.34), indicating support for the a posteriori model. The factor loadings for the five items to the ‘Realistic’ latent variable ranged from .50 to .81 (Mn = .63, SD = .14). Reliability estimate for the scale was .81.

#### Optimal best practice

Similar to that of RBP, we used a shorter version of the Optimal Outcome Questionnaire [57] to measure and assess the concept of OBP [20]. The five items included, for example: “I can achieve much more at university than I have indicated through my work so far” and “I want to learn and do more at university”. The goodness-of-fit index values of this model, Model M_1_, showed a relatively poor fit, as indicated by the following: χ^2^/d*f* = 17.35, *p* > .05, CFI = .80, TLI = .60, RMSEA = .13 (Lo90 = .11, Hi90 = .15), *p* < .001, and SRMR = .07. We respecified this a priori model with the inclusion of an error variance between Item 2 and Item 4. The goodness-of-fit index values for this a posteriori model, Model M_2_, showed an improvement in model fit: χ^2^/d*f* = 8.06, *p* < .001, CFI = .93, TLI = .83, RMSEA = .08 (Lo90 = .06, Hi90 = .11), *p* < .05, and SRMR = .04. The Δχ^2^ test between the two models was statistically significant, *p* < .001 (i.e., Δχ^2^_(Model M1 –Model M2)_ = 54.48), indicating support for the a posteriori model. To improve the fit further, we respecified Model M_2_ with the inclusion of an error variance between Item 2 and Item 3. The goodness-of-fit index values for this modified model, Model M_3_, improved over that of Model M_2_: χ^2^/d*f* = 4.92, *p* < .01, CFI = .97, TLI = .91, RMSEA = .06 (Lo90 = .03, Hi90 = .09), *p* > .05, and SRMR = .03. The Δχ^2^ test between the two models was statistically significant, *p* < .001 (i.e., Δχ^2^_(Model M1 –Model M2)_ = 17.50), indicating support for the a posteriori model. The factor loadings for the five items to the ‘Optimal’ latent variable ranged from .63 to .75 (Mn = .69, SD = .06). Reliability estimate for the scale was .79.

#### Personal resolve

We used five items [40] to measure and assess the concept of personal resolve. The items included, for example: “I will do whatever it takes to master my academic studies at university” and “I have a strong desire to succeed in my academic studies at university”. The goodness-of-fit index values showed a good model fit for this model, Model M_1_: χ^2^/d*f* = 7.32, *p* < .001, CFI = .98, TLI = .97, RMSEA = .07 (Lo90 = .05, Hi90 = .09), *p* < .05, and SRMR = .02. The factor loadings for the five items to the ‘Personal Resolve’ latent variable ranged from .60 to .78 (Mn = .74, SD = .07). Reliability estimate for the scale was .85.

#### Effective functioning

We used five items [40] to measure and assess the concept of effective functioning. The items included, for example: “I have been told at university that I am quite efficient’ and ‘I always keep to my routine when studying at university’. The goodness-of-fit index values showed a modest model fit for this model, Model M_1_: χ^2^/d*f* = 11.58, p < .001, CFI = .95, TLI = .89, RMSEA = .10 (Lo90 = .08, Hi90 = .13), *p* < .001, and SRMR = .04. We respecified this a priori model with the inclusion of an error variance between Item 4 and Item 5. The goodness-of-fit index values for this a posteriori model, Model M_2_, showed an improvement in model fit: χ^2^/d*f* = 6.49, *p* < .001, CFI = .98, TLI = .94, RMSEA = .07 (Lo90 = .05, Hi90 = .10), *p* > .05, and SRMR = .02. The Δχ^2^ test between the two models was statistically significant, *p* < .001 (i.e., Δχ^2^_(Model M1 –Model M2)_ = 32.00), indicating support for the a posteriori model. The factor loadings for the five items to the ‘Effective Functioning’ latent variable ranged from .47 to .65 (Mn = .58, SD = .07). Reliability estimate for the scale was .70.

#### Motivation towards academic learning

We adapted and used five items from the LOSO Questionnaire [11] to measure and assess the concept of motivation towards academic learning. The items included, for example: “I can do much better for some academic subjects at university than I do now” and “I rarely do my best at university”. A one-factor congeneric model was moderate in model fit, for example, as indicated from the goodness-of-fit index values: χ^2^/d*f* = 6.61, *p* < .001, CFI = .93, TLI = .87, RMSEA = .08 (Lo90 = .05, Hi90 = .10), *p* < .05, and SRMR = .04. An improvement in model fit was made with the inclusion of an error variance between Item 1 and Item 2. The goodness-of-fit index values for this model, Model M_2_, improved over that of Model M_1_’s: χ^2^/d*f* = 1.68, *p* > .05, CFI = .939 TLI = .98, RMSEA = .03 (Lo90 = .01, Hi90 = .06), *p* > .05, and SRMR = .02. Furthermore, a comparison of the two models, using the Δχ^2^ test (Δχ^2^_(Model M1 –Model M2)_ = 26.35), showed support for Model M_2_. The factor loadings for the five items to the ‘Motivation’ latent variable ranged from .60 to .78 (Mn = .69, SD = .07). Reliability estimate for the scale was .77.

#### Academic liking experience

We adapted five items from the Academic Well-Being Experience Questionnaire (SWBEQ)[58] to measure and assess the concept of academic liking experience. The items included, for example: “I really like going to university” and “I would rather stay at home than to attend university”. The goodness-of-fit index values showed a good model fit for this model, Model M_1_: χ^2^/d*f* = 5.91, *p* < .001, CFI = .97, TLI = .95, RMSEA = .07 (Lo90 = .05, Hi90 = .10), *p* > .05, and SRMR = .03. We respecified this a priori model with the inclusion of an error variance between Item 1 and Item 2. The goodness-of-fit index values for this a posteriori model, Model M_2_, showed an improvement in model fit: χ^2^/d*f* = 3.88, *p* < .01, CFI = .99, TLI = .97, RMSEA = .05 (Lo90 = .03, Hi90 = .08), *p* > .05, and SRMR = .02. The Δχ^2^ test between the two models was statistically significant, *p* < .001 (i.e., Δχ^2^_(Model M1 –Model M2)_ = 14.02), indicating support for the a posteriori model. The factor loadings for the five items to the ‘Liking’ latent variable ranged from .44 to .83 (Mn = .61, SD = .14). Reliability estimate for the scale was .83.

#### Emotional functioning

We adapted five items from the Academic Well-Being Experience Questionnaire (SWBEQ)[58] to measure and assess the concept of academic liking experience. The items included, for example: “I am always happy at university” and “My mood is always up at university”. The goodness-of-fit index values showed a good model fit for this model, Model M_1_: χ^2^/d*f* = 5.04, *p* < .001, CFI = .97, TLI = .95, RMSEA = .06 (Lo90 = .04, Hi90 = .09), *p* > .05, and SRMR = .03. We respecified this a priori model with the inclusion of an error variance between Item 4 and Item 5. The goodness-of-fit index values for this a posteriori model, Model M_2_, showed an improvement in model fit: χ^2^/d*f* = 2.85, *p* < .01, CFI = .99, TLI = .98, RMSEA = .04 (Lo90 = .02, Hi90 = .07), *p* > .05, and SRMR = .02. The Δχ^2^ test between the two models was statistically significant, *p* < .001 (i.e., Δχ^2^_(Model M1 –Model M2)_ = 13.78), indicating support for the a posteriori model. The factor loadings for the five items to the ‘Liking’ latent variable ranged from .47 to .69 (Mn = .60, SD = .10). Reliability estimate for the scale was .67.

#### Interest in learning tasks

We adapted and used five items from the LOSO Questionnaire [11] to measure and assess the concept of interest in learning tasks. The items included, for example: “I enjoy learning the different subjects in this university” and “I believe many things we learn in university are not important”. The initial a priori model, Model M_1_, showed a relatively modest fit of the data: χ^2^/d*f* = 5.75, *p* < .001, CFI = .98, TLI = .95, RMSEA = .07 (Lo90 = .05, Hi90 = .09), *p* > .05, and SRMR = .03. We respecified this a priori model with the inclusion of an error variance between Item 3 and Item 4. The goodness-of-fit index values for this a posteriori model, Model M_2_, showed an improvement in model fit: χ^2^/d*f* = 1.32, *p* < .01, CFI = .99, TLI = .97, RMSEA = .05 (Lo90 = .03, Hi90 = .08), *p* > .05, and SRMR = .02. The Δχ^2^ test between the two models was statistically significant, *p* < .001 (i.e., Δχ^2^_(Model M1 –Model M2)_ = 13.21), indicating support for the a posteriori model. The factor loadings for the five items to the ‘Liking’ latent variable ranged from .50 to .80 (Mn = .66, SD = .12). Reliability estimate for the scale was .82.

## Data analyses

We used SEM techniques [55, 56] to analyse the Taiwanese data. The technique of SEM is more rigorous than other multivariate statistical approaches for its acknowledgment of errors (i.e., E ≠ 0), and the use of both measurement and structural models [55, 56, 59] . SEM is advantageous as it allows a researcher to test and compare competing *a priori* models that have latent factors, measured indicators, and error specifications. It is also possible to refine an *a priori* model and alternative *a posteriori* models, using modification index (MI) values as a guide. What is important, however, is that SEM provides a basis for researchers to explore both *direct* and *indirect* effects, as well as yielding evidence for further development into mediating mechanisms of central variables [59–61].

We used the M*Plus* 8 statistical software package [62] with covariance matrices and maximum likelihood (ML) procedures to test the a priori model. We analysed covariance matrices because correlation matrix analysis is known to have problems, such as producing incorrect goodness-of-fit measures and standard errors [63, 64]. Furthermore, depending on the multivariate normality of the data, we selected to use one of the two estimation procedures–ML or robust ML (RML) procedures. ML procedure, for example, has been observed to perform reasonably well when data are normally distributed [65].

### SEM analyses: Comparison of competing models

Preliminary data analyses with SPSS 25 showed that the data were normally distributed–for example, the kurtosis and skewness values were within the range of ± 1.00, and there were no visible outliers. In our SEM approach, we considered two competing models for testing: (i) Model M_1_, which is the initial *a priori* model, as shown in Fig 1, and (ii) Model M_2_ is a respecification of Model M_1_, and is accordance with Baron and Kenny’s [66] criteria. Model M_1_ is relatively restricted and did not allow elaboration of decomposition of direct and indirect effects. For example, the indirect effect of effective functioning on motivation towards learning, via OBP was not determined, consequently because of the absence of the direct structural path from effective functioning to motivation towards learning. Model M_2_, in contrast, enabled examination of both indirect and potential mediating effects of variables, as we permitted the freeing of direct structural paths from effective functioning, emotional functioning, and personal resolve to motivation towards learning, interest in learning tasks, and academic liking experience–hence, as an example, Model M_2_ would allow us to explore the indirect effect of emotional functioning on academic liking experience, mediated in this case by OBP (i.e., there are three paths for examination: emotional functioning → OBP, OBP → academic liking experience, and emotional functioning → academic liking experience).

Model M_1_ was relatively sub-optimal in terms of model fit, as indicated by the following goodness-of-fit index values: χ^2^/d*f* = 2.93, *p* < .001, CFI = .89, TLI = .88, RMSEA = .044 (Lo90 = .042, Hi90 = .046), *p* > .05, and SRMR = .058. Model M_2_, in contrast, showed an improvement in the goodness-of-fit values: χ^2^/d*f* = 2.78, *p* < .001, CFI = .90, TLI = .90, RMSEA = .042 (Lo90 = .040, Hi90 = .044), *p* > .05, and SRMR = .052. The Δχ^2^ test between Model M_1_ and Model M_2_ was statistically significant, *p* < .001 (i.e., Δχ^2^_(Model M1 –Model M2)_(Δd*f* = 9) = 132.96), indicating support for the latter model–in other words, the results obtained affirm and support the inclusion of direct structural paths from effective functioning, emotional functioning, and personal resolve on motivation towards learning, interest in learning tasks, and academic liking experience. In social sciences research, it is not uncommon for researchers to find that some Likert-scale items have correlated errors between them; in other words, from a student’s point of view, there may be some commonality between two items or more and as such, the items are perceived as being similar to each other [63]. The modification fit indices (MIs) indicated a possible improvement in model fit of Model M_2_. This respecification of Model M_2_ was made with the inclusion of an error variance between Item 1 and Item 3 of the Motivation towards Academic Learning Subscale. The *a posteriori* model, Model M_3_, improved over that of Model M_2_ (e.g., χ^2^/d*f* = 2.69, *p* < .001, CFI = .92, TLI = .91, RMSEA = .041 (Lo90 = .039, Hi90 = .043), *p* > .05, and SRMR = .050. The Δχ^2^ test, likewise, produced a statistical significant difference (i.e., Δχ^2^_(Model M3 –Model M2)_ = 64.85).

**Fig 2:**
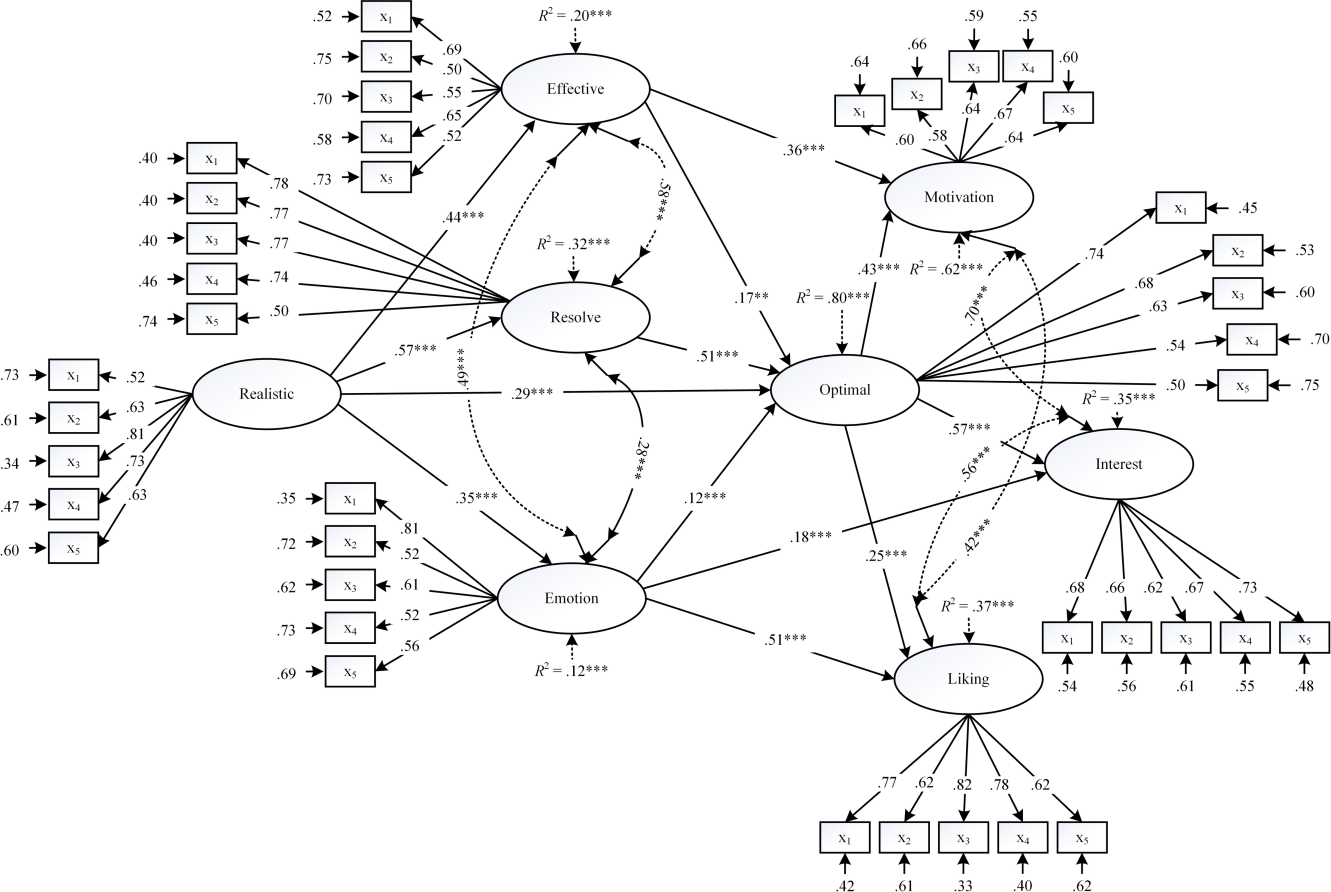
Solution of Model M_3_. Note: Realistic = realistic best practice, Optimal = optimal best practice, Effective = effective functioning, Resolve = personal resolve, Emotion = emotional functioning, Motivation = motivation towards learning, Interest = interest in learning tasks, Liking = academic liking experience. * p < .05, ** p < .01, *** p < .001.

A comparison of the three models shows that Model M_3_ is more superior in terms of model fit. We acknowledge that Model M_3_ is relatively complex and did not completely yield optimal goodness-of-fit index values (e.g., CFI = .92) as we would have liked. However, in totality, this model may be viewed as a basis for further inquiries. The results of the direct statistical significant paths from Model M_3_ are shown in Fig 2. An inspection of Model M_3_ shows that all 10 structural paths originally hypothesized in Model M_1_ are confirmed, with beta values ranging from .12, *p* < .001 (i.e., emotional functioning → OBP) to .57, *p* < .001 (i.e., RBP → personal resolve, OBP → interest in learning tasks). Furthermore, in relation to Model M_3_, which enabled us to identify indirect and mediating effects, three additional structural paths yielded statistical significance: effective functioning → motivation towards learning (β = .36, *p* < .001), emotional functioning → academic liking experience (β = .51, *p* < .001), and emotional functioning → interest in learning tasks (β = .18, *p* < .001). Notwithstanding the moderate model fit of the data, the evidence obtained fully supported our original conceptualization into the operational nature of optimal best.

### Direct, indirect, and total effects

The decomposition of effects is useful for the purpose of identifying statistical significant indirect effects and potential mediating mechanisms. Furthermore, a decomposition of indirect effects enables researchers to gauge into competing path trajectories and mediating variables–for example, the indirect effect of RBP on OBP may arise from three competing pathways: RBP on OBP, via effective functioning, RBP on OBP, via emotional functioning, and RBP on OBP, via personal resolve. In contrast, though, the indirect effect of effective functioning on motivation towards academic learning is mediated by OBP and no other variables. In a similar vein, the indirect effect of RBP on motivation towards academic learning may involve different competing pathways, for instance: RBP → OBP → motivation towards academic learning versus RBP → effective functioning → OBP → motivation towards academic learning *versus* RBP → emotional functioning → OBP → motivation towards academic learning *versus* RBP → personal resolve → OBP → motivation towards academic learning. Table 1 shows the decomposition of direct and indirect effects, whereas Table 2 summarizes the decomposition of the indirect effects. Finally, expanding Table 2, we present the potential mediating effects of the three psychological variables and OBP in Table 3.

**Table 1:** Decomposition of direct, indirect and total effects.

	Direct		Indirect		Total	
On Effective Functioning						
■ Of Realistic Best	.44	***	-		.44	***
On Emotional Functioning						
■ Of Realistic Best	.35	***	-		.35	***
On Personal Resolve						
■ Of Realistic Best	.57	***	-		.57	***
On Optimal Best						
■ Of Effective Functioning	.17	**	-		.17	**
■ Of Emotional Functioning	.12	***	-		.12	***
■ Of Personal Resolve	.50	***	-		.50	***
■ Of Realistic Best	.29	***	.40	***	.69	***
On Motivation Towards Learning						
■ Of Optimal Best	.43	***	-		.43	***
■ Of Effective Functioning	.36	***	.07	*	.43	***
■ Of Emotional Functioning	-.01		.05	**	.04	
■ Of Personal Resolve	.07		.22	***	.29	***
■ Of Realistic Best	-		.49	***	.49	***
On Academic Liking Experience						
■ Of Optimal Best	.25	**	-		.25	**
■ Of Effective Functioning	-.12		.04	*	-.08	
■ Of Emotional Functioning	.51	***	.03	*	.54	***
■ Of Personal Resolve	.02		.12	**	.14	**
■ Of Realistic Best	-		.31	***	.31	***
On Interest in Learning Tasks						
■ Of Optimal Best	.57	***	-		.57	***
■ Of Effective Functioning	-.05		.10	*	.05	
■ Of Emotional Functioning	.18	***	.07	**	.25	***
■ Of Personal Resolve	-.07		.29	***	.22	***
■ Of Realistic Best	-		.39	***	.39	***

Note

* *p* < .05

** *p* < .01

*** *p* < .001.

**Table 2:** Decomposition of indirect effects.

Predictor			Outcome	*β*	*p*
Realistic Best	Effective Functioning		Optimal Best	.07	**
Realistic Best	Emotional Functioning		Optimal Best	.04	**
Realistic Best	Personal Resolve		Optimal Best	.29	***
Effective Functioning	Optimal Best		Motivation Towards Learning	.07	*
Emotional Functioning	Optimal Best		Motivation Towards Learning	.05	*
Personal Resolve	Optimal Best		Motivation Towards Learning	.22	***
Realistic Best	Optimal Best		Motivation Towards Learning	.12	***
Realistic Best	Effective Functioning		Motivation Towards Learning	.16	***
Realistic Best	Emotional Functioning		Motivation Towards Learning	-.00	
Realistic Best	Personal Resolve		Motivation Towards Learning	.04	
Realistic Best	Effective Functioning	Optimal Best	Motivation Towards Learning	.03	*
Realistic Best	Emotional Functioning	Optimal Best	Motivation Towards Learning	.02	*
Realistic Best	Personal Resolve	Optimal Best	Motivation Towards Learning	.12	***
Effective Functioning	Optimal Best		Interest in Learning Tasks	.10	*
Emotional Functioning	Optimal Best		Interest in Learning Tasks	.07	**
Personal Resolve	Optimal Best		Interest in Learning Tasks	.29	***
Realistic Best	Optimal Best		Interest in Learning Tasks	.16	***
Realistic Best	Effective Functioning		Interest in Learning Tasks	-.02	
Realistic Best	Emotional Functioning		Interest in Learning Tasks	.06	***
Realistic Best	Personal Resolve		Interest in Learning Tasks	-.04	
Realistic Best	Effective Functioning	Optimal Best	Interest in Learning Tasks	.04	*
Realistic Best	Emotional Functioning	Optimal Best	Interest in Learning Tasks	.02	**
Realistic Best	Personal Resolve	Optimal Best	Interest in Learning Tasks	.16	***
Effective Functioning	Optimal Best		Academic Liking Experience	.04	*
Emotional Functioning	Optimal Best		Academic Liking Experience	.03	*
Personal Resolve	Optimal Best		Academic Liking Experience	.12	**
Realistic Best	Optimal Best		Academic Liking Experience	.07	**
Realistic Best	Effective Functioning		Academic Liking Experience	-.05	
Realistic Best	Emotional Functioning		Academic Liking Experience	.18	***
Realistic Best	Personal Resolve		Academic Liking Experience	.01	
Realistic Best	Effective Functioning	Optimal Best	Academic Liking Experience	.02	*
Realistic Best	Emotional Functioning	Optimal Best	Academic Liking Experience	.01	*

Note

* *p* < .05

** *p* < .01

*** *p* < .001.

**Table 3:** Mediating effects.

Predictor		Mediator		Outcome	*β*	*p*
Realistic Best		Effective Functioning		Optimal Best	.07	**
Realistic Best		Emotional Functioning		Optimal Best	.04	**
Realistic Best		Personal Resolve		Optimal Best	.29	***
Effective Functioning		Optimal Best		Motivation Towards Learning	.07	*
Emotional Functioning		Optimal Best		Motivation Towards Learning	.05	**
Personal Resolve		Optimal Best		Motivation Towards Learning	.22	***
Realistic Best		Optimal Best		Motivation Towards Learning	.30	***
	Realistic Best	Optimal Best		Motivation Towards Learning	.12	***
	Realistic Best	Effective Functioning	Optimal Best	Motivation Towards Learning	.03	*
	Realistic Best	Emotional Functioning	Optimal Best	Motivation Towards Learning	.02	*
	Realistic Best	Personal Resolve	Optimal Best	Motivation Towards Learning	.12	***
Effective Functioning		Optimal Best		Interest in Learning Tasks	.10	*
Emotional Functioning		Optimal Best		Interest in Learning Tasks	.07	**
Personal Resolve		Optimal Best		Interest in Learning Tasks	.29	***
Realistic Best		Optimal Best		Interest in Learning Tasks	.39	***
	Realistic Best	Optimal Best		Interest in Learning Tasks	.16	***
	Realistic Best	Effective Functioning	Optimal Best	Interest in Learning Tasks	.04	*
	Realistic Best	Emotional Functioning	Optimal Best	Interest in Learning Tasks	.02	**
	Realistic Best	Personal Resolve	Optimal Best	Interest in Learning Tasks	.16	***
Effective Functioning		Optimal Best		Academic Liking Experience	.04	*
Emotional Functioning		Optimal Best		Academic Liking Experience	.03	*
Personal Resolve		Optimal Best		Academic Liking Experience	.12	**
Realistic Best		Optimal Best		Academic Liking Experience	.17	**
	Realistic Best	Optimal Best		Academic Liking Experience	.07	**
	Realistic Best	Effective Functioning	Optimal Best	Academic Liking Experience	.02	*
	Realistic Best	Emotional Functioning	Optimal Best	Academic Liking Experience	.01	*
	Realistic Best	Personal Resolve	Optimal Best	Academic Liking Experience	.07	**

Note:

* *p* < .05

** *p* < .01

*** *p* < .001.

An inspection of Tables 1, 2 and 3 shows empirical support for the potential mediating roles of the following variables: (i) effective functioning (e.g., RBP → effective functioning → OBP: β = .07, *p* < .001), (ii) emotional functioning (e.g., RBP → emotional functioning → OBP: β = .04, *p* < .01), (iii) personal resolve (e.g., RBP → effective functioning → OBP: β = .29, *p* < .001), and (iv) OBP (e.g., effective functioning → OBP → motivation towards academic learning: β = .07, *p* < .05). Furthermore, from Table 3, we identified the following statistical significant pathways that originated from RBP to the different educational outcomes, via the three psychological variables *and* OBP–for example: (i) RBP → effective functioning → OBP → motivation towards academic learning (β = .03, *p* < .05), RBP → emotional functioning → OBP → motivation towards academic learning (β = .02, *p* < .05), and RBP → personal resolve → OBP → motivation towards academic learning (β = .12, *p* < .001), (ii) RBP → effective functioning → OBP → interest in learning tasks (β = .04, *p* < .05), RBP → emotional functioning → OBP → interest in learning tasks (β = .02, *p* < .05), and RBP → personal resolve → OBP → interest in learning tasks (β = .16, *p* < .001), and (iii) RBP → effective functioning → OBP → academic liking experience (β = .02, *p* < .05), RBP → emotional functioning → OBP → academic liking experience (β = .01, *p* < .05), and RBP → personal resolve → OBP → academic liking experience (β = .07, *p* < .01).

It is important that interpret the aforementioned results into the mediating roles of the three psychological variables and OBP with caution. Extensive progress, both in terms of conceptualization and empirical research development, has been made since the seminal publication of Baron and Kenny’s [66] article on mediating and moderating effects. Determination of mediating effects is limited when cross-sectional and non-experimental data are used [60, 61, 67]. What we need to consider, in this analysis, is the fulfilment of two main criteria, namely: (i) a need for sequencing of the variables (i.e., determinant → mediator → outcome), which implies *time precedence*, and (ii) a need to establish causal flow (e.g., determinant → mediator), which implies the use of *experimental treatments*. On this basis, we acknowledge our use of non-experimental data across one time point as major caveat that limits us from making sound inference of mediating effects.

## Discussion of results

The study of optimal best has evolved over the years to include different theorizations and conceptualizations from researchers in the field of psychology [3, 9, 10, 12]. One major emphasis, in particular, has examined the operational nature and characteristics of a person’s best practice–for example, how does a person achieve a state of optimal best? This inquiry, we contend, has potential wide-ranging implications in the areas of education, health, sports, etc. In this analysis, a focus on a person’s achievement of optimal best places emphasis on the capitalization and use of appropriate human resources.

Our research investigation, drawn from a correlational approach, addressed two fundamental related inquiries: (i) the extent to which RBP would act as a determinant and different psychological variables (e.g., effective functioning) act as determinants and potential mediators of OBP, and (ii) the potential influence of an internal state of OBP on different types of educational outcomes. We used SEM techniques to test and affirm an *a priori* model that focused on the following: the direct contributions of comparable psychological variables in the account and explanation of OBP, and the potential effect of a state of OBP on different types of adaptive outcomes. We confirm that, overall, the results obtained fully supported our original hypothesis. This evidence, which we discuss in this section of the article, is substantive in terms of its empirical and methodological contributions. In light of this research development, we consider the synergy of undertaking a research inquiry into OBP using more complex methodological designs noteworthy.

### Account and explanation of OBP

The concept of OBP, consistent with Martin’s [12, 13] work and other researchers’ inquiries [8, 10, 40], is interesting for its emphasis on the *maximization in experience of a person’s functioning*. Achievement of OBP in a subject matter is a central feat of a person’s learning experience, situated within different educational and/or non-educational contexts. What is of interest for us then, as educators, is how do we encourage and ensure that students reach optimal best levels? Furthermore, of interest for us to consider is the potential effect of OBP on different types of adaptive outcomes, such as academic performance in school settings. We contend that our focus of inquiry, which attempted to address this topical theme, has relevance to other non-academic types of functioning–for example, a person’s striving to achieve an optimal level of happiness after recent setbacks.

From our results, we note that a person’s current knowledge base makes a major contribution in the prediction of his/her OBP. Self-awareness of current competence in a subject matter, in this analysis, may a person to consider and project his/her optimal best–for example, what is the best that I can do in Calculus given what I know, at present? This finding supports our innovative theoretical positioning–namely, a student’s self-awareness and indication of his/her *present state* of functioning (e.g., academic competence in mathematics) may serve as a *source of personal reflection*, which could then motivate and predict his/her maximized level of functioning. Our theorization, in this case supported by the statistical significant effect of RBP on OBP, recognizes the capitalization of a person’s existing level of knowledge and experience. In the context of schooling, a student’s current knowledge base may function as a *personal point of reference* for aspiration, motivation, and achievement. A low-to-moderate level of RB, for example, may demotivate and undermine a student’s capability. A high level of RB, in contrast, may aspire and motivate a student to achieve a level of exceptionality.

The validation of relationship between RBP and OBP, from our point of view, is insightful for the purpose of educational practice, which may involve the use of *verbal discourse strategies* (e.g., effort feedback)[68, 69]) to encourage students to strive for maximization in learning outcomes. Providing a student with timely feedback could help to inform him/her of his/her academic progress in a subject matter. From an educational point of view, we contend that a teacher’s accurate assessment and fair reporting of a student’s current academic capability level could help to inform him/her of a corresponding level of best practice for achievement. Inaccurate judgments (e.g., the use of incredulous feedbacks), which Pajares [70] coins as *miscalibration*, may produce detrimental consequences. A teacher’s misjudgement of a student’s capability, for example, may instil false hope and/or expectation, which would then lower his/her sense of confidence.

At the same time, aside from the direct impact of RBP, we found that effective functioning, emotional functioning, and personal resolve accounted and explained the achievement of OBP. A focus on organization, structured thinking and behaviour and efficiency, in this analysis, may assist a student to achieve OBP. At the same time, in tandem with engagement in effective functioning, the student’s state of resolve and decisiveness to learn may facilitate and enable the striving of OBP. Personal resolve in a subject matter that is of interest, in this sense, could serve as a source of persistence and effort expenditure. In a similar vein, as noted, positive emotional functioning (e.g., happiness) is associated with OBP. This evidence affirms the importance of the operational nature of positive emotions, in particular. Negative emotional functioning (e.g., a heightened state of sadness), in contrast, is likely to deter a student’s progress from his/her knowledge base.

The first aspect of the present study has produced evidence that is consistent with our original hypothesis and, in particular, with existing theorizations and research development into the topical theme of optimal functioning. We have, in this case, provided clarity into the achievement of personal best [9, 10, 39]. In the absence of experimental treatments, researchers could consider non-experimental predictive effects (e.g., effective functioning → OBP, β = .17, *p* < .01) as a proxy index of ‘optimizing effects’. From our point of view, the pattern in associations established in this study has formed a basis for further inquiries. One notable focus is to develop appropriate methodological approaches that could measure and assess the essence and magnitude of optimizing effects [9, 10].

### The positive effect of OBP on adaptive outcomes

Researchers have postulated that personal experience of OBP could positively influence different types of adaptive outcomes. This proposition reflects the paradigm of positive psychology [1, 2], and highlights the educational potency of a student’s personal best. In their recent longitudinal research, for example, G.A.D. Liem et al. [8] found from autoregressive analyses that personal best sustained its influence on different achievement-related outcomes across time: deep learning, academic flow, and teacher relationship. In another longitudinal study, similarly, Martin and Liem [19] reported the effect of personal best on academic engagement and achievement outcome. Hence from this evidence, we contend that experience of OBP could have enriched and positive consequences for students.

The present study has found similar findings to those of previous research [8, 19], highlighting the potent effect of OBP in educational contexts. This evidence emphasizes the fact that OBP could serve as an important source of information to produce a number of positive yields, such as enhancing a student’s interest in learning tasks and a state of motivation toward academic learning. Effective functioning, likewise, is analogously associated with a state of motivation towards academic learning. The importance of efficiency, structured thinking, organization, and purposive intent may guide and motivate a student’s behaviour for effective learning.

A student’s OBP, from this affirmation, is noted to account for a substantial amount of variance in his/her academic liking experience. Students who do well and achieve optimal levels of capability are more likely to report their academic liking of university. This finding supports existing theorizations and, again, emphasizes the positive characteristics of OBP–for example, successful achievement of OBP may instil a ‘feel-good’ experience and belief that anything in academia is possible. An issue for us to consider then, is whether a student’s inability to achieve OBP would negate his/her academic liking experience at university. Does stagnation in learning for a subject matter or a suit of subjects, consequently as a result of conflicting interests and/or influences contribute to a student’s negative academic liking experience? At the same time, we found that positive emotional functioning served as an important antecedent of a student’s academic liking experience and personal interest in learning tasks at university.

In summary, the preceding section has explored the potent effect of OBP on different types of educational outcomes. This evidence, in its totality, has a number of educational implications for consideration. As educators, we need to explore pathways (e.g., interesting subject content), pedagogical strategies, and/or educational programs that could encourage students to strive for optimal levels of academic experience in their schooling. As we note from the present study (e.g., Table 3: mediating effects), OBP is a central concept that operates as both an outcome and a determinant of different achievement-related outcomes. Purposive implementation of *in situ* experimental treatments that focus on the *heightening* of OBP (e.g., the use of strategies (e.g., planning) to engage in effective functioning) could produce improvement in different types of educational outcomes. At the same time, from the present study (e.g., Fig 2), the potent effect of RBP also recognizes the saliency of a person’s current knowledge base. We argue that in the context of effective teaching and learning, it would be appropriate for both educators and students to place strong emphasis on *self-awareness and self-reflection of current capability*–for example, what can I do, at present, and how can I capitalize on my current knowledge to assist in the achievement of OBP?

## Caveats and future research

The present study has introduced an important topical theme for research development. Notwithstanding the substantive empirical contribution that we have made, there are a number of notable caveats that warrant continuing research. We recognize that the use of cross-sectional non-experimental data is extremely limited, especially when one wishes to explore causal flows [71, 72] and/or mediating effects [60, 61]. Researchers have inferred and concluded the mediating mechanisms of different psychological and educational variables using cross-sectional data [73–75]. Likewise, from the present study, we have alluded to the decomposition of indirect effects and the potential evidence of mediating mechanisms of OBP and the three psychological variables. However, from Baron and Kenny’s [66] work and more recent progress, we acknowledge that true mediating effects are determined when two major criteria are met [60, 67, 76]: (i) the establishment of causal flows (i.e., A → B, where → = causal effect) and, in this case, involves the use of an *experimental treatment*, and (ii) the establishment of sequencing, which involves the *precedence of time differences* (i.e., T_1_, T_2_, T_3_, etc.). Hence, in this sense, we acknowledge that our research investigation is somewhat limited in its use of cross-sectional data, which do not permit us to test an alternative conceptualization, namely, the potential influence of OBP on effective functioning, emotional functioning, and personal resolve. As a possibility, for example, we could measure and assess RBP at T_1_, OBP at T_3_, effective functioning, emotional functioning, and personal resolve at T_2_ and T_4_, and motivation towards learning, interest in learning tasks, and academic liking experience at T_5_. This methodological consideration is innovative as it would enable researchers to explore the ‘cause and predictor’ of OBP (e.g., the effect of T_2_ emotional functioning on T_3_ OBP *versus* the effect of T_3_ OBP on T_4_ emotional functioning). We acknowledge that personal experience of an optimal level of best practice could, in fact, yield positive psychological and educational outcomes. However, having said this, per our original conceptualization, we hypothesized that *enactment of psychological variables* (e.g., effective functioning) would optimize a person’s best practice.

The study of personal best [3, 9, 10, 13], in its totality, encompasses a number of theoretical aspects and research inquiries, which we have previously discussed–for example, the notion of OBP and the ‘optimizing’ effect of a psychological variable. One fundamental issue that is extremely complex concerns the *measurement and assessment of optimal best*. Research investigations to date, including the present study, have predominantly used correlational analyses derived from non-experimental data to infer the effectiveness of an optimizing variable [8, 29]. Associations and predictive effects (e.g., β value), in this sense, do not adequately explain the ‘magnitude’ of an optimizing effect of a psychological or educational variable. In a similar vein, indication of optimal best has relied on the use of surveys and Likert-scale measures. A composite score, in this analysis, may provide information regarding a person’s level of best practice [29]. This methodological approach, we argue, is more effective as a *proxy* measure of a person’s optimal best.

It is advisable for researchers to consider alternative methodological approaches that are more robust, and could accurately measure and assess OBP. Of interest, in this case, is the development of an appropriate *quantitative metric* that could equate to a person’s optimal best.

This tenet, we contend, considers the transformation of the RBP-OBP difference [9, 10] into an ‘optimizing equivalency’ or ‘index’. In the area of *cognitive load theory* [77, 78] there is a theoretical concept, known as the *index of cognitive complexity*, which is a numerical value that reflects a person’s indication of his/her perception of the difficulty and complexity of a learning task. We propose similarly an *index of optimization* (i.e., denoted as ‘IO’), which could vary in numerical values, and intricately associate with the index of cognitive complexity. The index of optimization, for us, could define and reflect the *magnitude* (i.e., strength) of the process of optimization–for example, an optimal learning task that is relatively complex would require ‘more’ magnitude of optimization.

Finally, in relation to our findings, we contend that the RBP-OBP difference is an interesting theoretical concept for advancement and development. The RBP-OBP difference, which we conceptualized as an internal state of flourishing, is relatively difficult to measure and assess. So far, from the present study, we have used the Optimal Outcome Questionnaire [57] to measure and assess the two levels of best practice. This quantitative methodological design is viable and has produced evidence that showed the association between RBP and OBP. However, a non-experimental methodology is somewhat limited and does not provide in-depth understanding of the operational process of *how* effective functioning, as a psychological mechanism, say, actually enables a person to achieve OBP from RBP. Furthermore, in relation to our previous mentioning of the index of optimization, we propose an ‘optimizing effect’ of a psychological variable, coined as ‘γ’ in terms of scientific notation, which could intricately associate with the RB-OBP difference. As a point of summary for possible development, consider the derivative of the index of optimization as a combination of the RBP-OPB difference (i.e., Δ_(OBP-RBP)_) and the optimizing effect (i.e., γ)–that is: Index of Optimization (IO) = Δ_(OBP-RBP)_ × γ, where γ = optimizing effect. What this entails then, from our conceptualization, is that the optimizing effect of a psychological variable (e.g., effective functioning) could vary in its magnitude or strength and, more importantly, it is plausible for us to ‘quantify’ the index of optimization. A standardized numerical value that is small, for example, would indicate a minimal level of magnitude of optimization, whereas a larger numerical value would imply a higher level of magnitude of optimization.

## Conclusion

In conclusion, the study of optimal best has consisted of different theorizations, conceptualizations, and research inquiries. Optimal best, reflecting the paradigm of positive psychology [1, 2], is a central feat of human agency. Personal experience of optimal best plays an important role in helping a person to flourish, both educationally and non-educationally. Our correlational research, overall, provided substantive evidence to support further development into the concept of optimal best. In particular, unlike previous considerations and from Fraillon’s [10] seminal publication, we proposed a point of reference by a person could consider his/her optimal best level. This postulation, which we coined as the RBP-OBP difference, enabled us to explore the operational nature of optimal best.

## Supporting information

S1 Table (Covariance and correlation matrixes). (PDF)
