# Supplementary material for: Understanding levels of best practice: An empirical validation
Source: PLoS One. 2018 Jun 14;13(6):e0198888. doi: 10.1371/journal.pone.0198888 (PMC6002041; doi:10.1371/journal.pone.0198888)
Supplement: S1 Table — (PDF) [file pone.0198888.s001.pdf]

Covariances

	RB4	RB5	RB6	RB7	RB8
RB4	0.690				
RB5	0.284	0.796			
RB6	0.261	0.378	0.681		
RB7	0.318	0.350	0.452	0.839	
RB8	0.306	0.303	0.369	0.532	0.843
OB2	0.151	0.204	0.283	0.250	0.258
OB4	0.143	0.188	0.234	0.221	0.245
OB6	0.108	0.190	0.223	0.199	0.210
OB7	0.096	0.183	0.190	0.174	0.200
OB8	0.030	0.186	0.162	0.117	0.147
EF1	0.111	0.127	0.194	0.185	0.153
EF3	-0.040	-0.016	-0.002	0.030	0.046
EF4	0.061	0.108	0.094	0.137	0.108
EF5	0.081	0.106	0.116	0.123	0.138
EF6	0.077	0.122	0.113	0.141	0.161
EW1	0.031	0.170	0.156	0.110	0.120
EW4	0.000	0.106	0.122	0.068	0.044
EW6	0.070	0.250	0.175	0.183	0.162
EW3	0.052	0.114	0.038	0.129	0.063
EW8	0.037	0.114	0.128	0.069	0.098
PR1	0.111	0.166	0.248	0.269	0.229
PR2	0.126	0.177	0.238	0.206	0.168
PR3	0.128	0.184	0.244	0.189	0.205
PR4	0.065	0.126	0.201	0.181	0.152
PR6	0.111	0.108	0.182	0.103	0.141
MTL1	0.109	0.107	0.162	0.156	0.156
MTL5	0.082	0.083	0.183	0.127	0.138
MTL7	0.130	0.119	0.173	0.172	0.161

MTL9	0.117	0.125	0.169	0.177	0.144
MTL10	0.176	0.208	0.231	0.247	0.220
IL2	0.129	0.176	0.217	0.184	0.178
IL3	0.094	0.100	0.131	0.104	0.096
IL6	0.054	0.098	0.139	0.088	0.092
IL7	0.135	0.125	0.191	0.160	0.144
IL10	0.148	0.180	0.198	0.217	0.168
LAE1	0.037	0.126	0.139	0.116	0.089
LAE3	0.026	0.069	0.067	0.068	0.041
LAE4	0.031	0.130	0.112	0.105	0.095
LAE5	0.065	0.117	0.124	0.128	0.109
LAE2	0.180	0.196	0.207	0.211	0.181

Covariances

	OB2	OB4	OB6	OB7	OB8	
OB2	0.557					
OB4	0.292	0.544				
OB6	0.286	0.253	0.691			
OB7	0.241	0.278	0.343	0.757		
OB8	0.189	0.220	0.309	0.418	0.759	
EF1	0.205	0.184	0.231	0.207	0.210	
EF3	0.029	0.010	0.099	0.051	0.148	
EF4	0.157	0.134	0.155	0.155	0.146	
EF5	0.197	0.177	0.167	0.148	0.130	
EF6	0.145	0.188	0.153	0.215	0.236	
EW1	0.122	0.163	0.232	0.216	0.313	
EW4	0.116	0.115	0.116	0.124	0.131	
EW6	0.122	0.153	0.208	0.211	0.266	
EW3	-0.001	0.050	0.078	0.069	0.152	

EW8	0.119	0.094	0.162	0.139	0.160
PR1	0.318	0.266	0.266	0.240	0.258
PR2	0.277	0.221	0.196	0.171	0.145
PR3	0.271	0.245	0.234	0.214	0.176
PR4	0.237	0.217	0.248	0.197	0.219
PR6	0.220	0.148	0.246	0.153	0.192
MTL1	0.182	0.182	0.150	0.140	0.127
MTL5	0.197	0.122	0.220	0.144	0.114
MTL7	0.197	0.147	0.185	0.178	0.140
MTL9	0.229	0.155	0.182	0.148	0.130
MTL10	0.249	0.227	0.188	0.137	0.140
IL2	0.194	0.150	0.222	0.134	0.127
IL3	0.173	0.140	0.175	0.144	0.155
IL6	0.153	0.112	0.191	0.152	0.134
IL7	0.135	0.123	0.177	0.118	0.134
IL10	0.190	0.141	0.184	0.143	0.155
LAE1	0.135	0.112	0.149	0.142	0.196
LAE3	0.078	0.061	0.140	0.113	0.142
LAE4	0.123	0.102	0.147	0.136	0.185
LAE5	0.125	0.104	0.176	0.149	0.163
LAE2	0.189	0.132	0.199	0.150	0.160

Covariances

	EF1	EF3	EF4	EF5	EF6
EF1	0.581				
EF3	0.169	0.920			
EF4	0.211	0.197	0.599		
EF5	0.239	0.128	0.342	0.550	
EF6	0.239	0.215	0.220	0.265	0.859

EW1	0.194	0.128	0.150	0.163	0.229
EW4	0.149	0.028	0.094	0.121	0.130
EW6	0.161	0.147	0.114	0.135	0.182
EW3	0.047	0.168	0.044	0.052	0.123
EW8	0.163	0.081	0.104	0.146	0.141
PR1	0.261	0.087	0.167	0.220	0.191
PR2	0.193	0.031	0.150	0.186	0.113
PR3	0.205	0.068	0.158	0.187	0.139
PR4	0.235	0.119	0.158	0.203	0.178
PR6	0.220	0.155	0.097	0.145	0.115
MTL1	0.175	0.014	0.142	0.152	0.103
MTL5	0.287	0.078	0.169	0.182	0.150
MTL7	0.212	0.131	0.198	0.200	0.168
MTL9	0.198	0.044	0.148	0.178	0.133
MTL10	0.179	-0.024	0.138	0.175	0.117
IL2	0.177	0.028	0.096	0.132	0.083
IL3	0.143	0.072	0.111	0.127	0.095
IL6	0.130	0.070	0.146	0.127	0.065
IL7	0.115	0.012	0.110	0.096	0.017
IL10	0.116	0.064	0.110	0.095	0.063
LAE1	0.101	0.067	0.073	0.071	0.109
LAE3	0.087	0.058	0.097	0.083	0.070
LAE4	0.093	0.024	0.097	0.103	0.098
LAE5	0.148	0.102	0.101	0.101	0.107
LAE2	0.158	0.039	0.066	0.103	0.070

Covariances

	EW1	EW4	EW6	EW3	EW8
EW1	0.689				

EW4	0.274	0.580			
EW6	0.423	0.182	0.964		
EW3	0.254	0.033	0.506	1.053	
EW8	0.319	0.288	0.243	0.183	0.677
PR1	0.168	0.129	0.137	0.038	0.147
PR2	0.105	0.103	0.074	0.002	0.116
PR3	0.158	0.112	0.155	0.070	0.159
PR4	0.173	0.119	0.129	0.092	0.181
PR6	0.120	0.090	0.138	0.055	0.132
MTL1	0.142	0.129	0.097	-0.018	0.155
MTL5	0.136	0.132	0.091	-0.029	0.139
MTL7	0.137	0.091	0.104	0.033	0.087
MTL9	0.142	0.133	0.109	-0.011	0.154
MTL10	0.121	0.137	0.188	0.043	0.115
IL2	0.147	0.148	0.195	0.085	0.140
IL3	0.158	0.139	0.167	0.080	0.132
IL6	0.170	0.124	0.196	0.085	0.160
IL7	0.092	0.113	0.191	0.106	0.074
IL10	0.117	0.121	0.196	0.115	0.085
LAE1	0.238	0.174	0.262	0.159	0.169
LAE3	0.185	0.142	0.173	0.092	0.117
LAE4	0.233	0.201	0.225	0.066	0.185
LAE5	0.198	0.161	0.180	0.069	0.152
LAE2	0.182	0.141	0.327	0.183	0.143

Covariances

	PR1	PR2	PR3	PR4	PR6
PR1	0.687				
PR2	0.344	0.462			

PR3	0.338	0.308	0.530		
PR4	0.386	0.287	0.330	0.582	
PR6	0.260	0.198	0.225	0.230	0.755
MTL1	0.179	0.160	0.181	0.144	0.169
MTL5	0.222	0.175	0.192	0.192	0.216
MTL7	0.207	0.146	0.185	0.158	0.185
MTL9	0.225	0.166	0.203	0.177	0.216
MTL10	0.217	0.186	0.186	0.161	0.210
IL2	0.171	0.127	0.185	0.181	0.277
IL3	0.145	0.115	0.134	0.130	0.216
IL6	0.134	0.087	0.131	0.131	0.245
IL7	0.145	0.088	0.099	0.126	0.266
IL10	0.172	0.107	0.133	0.098	0.235
LAE1	0.155	0.074	0.115	0.105	0.172
LAE3	0.096	0.076	0.112	0.068	0.107
LAE4	0.136	0.097	0.121	0.097	0.098
LAE5	0.151	0.082	0.118	0.132	0.158
LAE2	0.187	0.106	0.146	0.145	0.237

Covariances

	MTL1	MTL5	MTL7	MTL9	MTL10
MTL1	0.458				
MTL5	0.217	0.659			
MTL7	0.194	0.258	0.618		
MTL9	0.194	0.237	0.309	0.594	
MTL10	0.214	0.222	0.231	0.268	0.670
IL2	0.179	0.213	0.191	0.240	0.315
IL3	0.155	0.210	0.224	0.225	0.210
IL6	0.156	0.188	0.211	0.181	0.199

IL7	0.179	0.182	0.154	0.171	0.283
IL10	0.176	0.190	0.230	0.231	0.306
LAE1	0.134	0.127	0.158	0.182	0.159
LAE3	0.084	0.088	0.103	0.139	0.094
LAE4	0.131	0.112	0.142	0.192	0.148
LAE5	0.148	0.142	0.201	0.204	0.160
LAE2	0.145	0.112	0.196	0.195	0.314

Covariances

	IL2	IL3	IL6	IL7	IL10
IL2	0.714				
IL3	0.257	0.564			
IL6	0.281	0.330	0.660		
IL7	0.381	0.277	0.311	0.763	
IL10	0.350	0.299	0.294	0.384	0.747
LAE1	0.207	0.255	0.262	0.207	0.292
LAE3	0.109	0.162	0.153	0.103	0.182
LAE4	0.174	0.194	0.172	0.166	0.234
LAE5	0.205	0.219	0.246	0.207	0.274
LAE2	0.334	0.273	0.285	0.338	0.374

Covariances

	LAE1	LAE3	LAE4	LAE5	LAE2
LAE1	0.685				
LAE3	0.317	0.562			
LAE4	0.388	0.356	0.556		
LAE5	0.357	0.279	0.373	0.560	

LAE2	0.395	0.264	0.274	0.323	1.009

Correlations

	RB4	RB5	RB6	RB7	RB8
RB4	1.000				
RB5	0.383	1.000			
RB6	0.381	0.513	1.000		
RB7	0.417	0.428	0.598	1.000	
RB8	0.401	0.369	0.487	0.632	1.000
OB2	0.244	0.307	0.460	0.365	0.377
OB4	0.233	0.285	0.385	0.327	0.362
OB6	0.156	0.256	0.325	0.261	0.275
OB7	0.133	0.235	0.265	0.218	0.251
OB8	0.042	0.240	0.225	0.146	0.184
EF1	0.176	0.186	0.308	0.265	0.218
EF3	-0.050	-0.019	-0.003	0.034	0.053
EF4	0.095	0.157	0.147	0.193	0.152
EF5	0.131	0.160	0.190	0.181	0.203
EF6	0.100	0.147	0.148	0.166	0.189
EW1	0.045	0.230	0.228	0.145	0.158
EW4	0.000	0.155	0.194	0.098	0.062
EW6	0.086	0.285	0.216	0.203	0.179
EW3	0.061	0.124	0.045	0.137	0.067
EW8	0.055	0.156	0.189	0.092	0.130
PR1	0.161	0.224	0.362	0.354	0.300
PR2	0.224	0.291	0.424	0.331	0.269
PR3	0.212	0.283	0.406	0.284	0.306
PR4	0.102	0.185	0.320	0.259	0.216
PR6	0.154	0.140	0.253	0.129	0.177

MTL1	0.194	0.177	0.290	0.252	0.252
MTL5	0.122	0.114	0.273	0.171	0.184
MTL7	0.199	0.170	0.266	0.239	0.223
MTL9	0.182	0.181	0.266	0.251	0.204
MTL10	0.259	0.285	0.342	0.329	0.293
IL2	0.184	0.234	0.311	0.238	0.229
IL3	0.150	0.150	0.211	0.151	0.140
IL6	0.080	0.135	0.208	0.118	0.123
IL7	0.186	0.160	0.265	0.200	0.179
IL10	0.207	0.233	0.278	0.274	0.211
LAE1	0.054	0.171	0.203	0.153	0.116
LAE3	0.042	0.104	0.108	0.099	0.060
LAE4	0.050	0.195	0.182	0.154	0.138
LAE5	0.105	0.175	0.201	0.187	0.159
LAE2	0.215	0.218	0.249	0.229	0.196

Correlations

	OB2	OB4	OB6	OB7	OB8
OB2	1.000				
OB4	0.530	1.000			
OB6	0.462	0.413	1.000		
OB7	0.371	0.434	0.474	1.000	
OB8	0.291	0.342	0.426	0.551	1.000
EF1	0.361	0.327	0.365	0.312	0.316
EF3	0.040	0.015	0.124	0.061	0.177
EF4	0.272	0.235	0.242	0.230	0.216
EF5	0.355	0.323	0.271	0.229	0.201
EF6	0.209	0.274	0.198	0.267	0.292
EW1	0.197	0.266	0.337	0.300	0.433

EW4	0.204	0.205	0.183	0.187	0.198
EW6	0.167	0.211	0.254	0.247	0.311
EW3	-0.001	0.066	0.091	0.077	0.170
EW8	0.194	0.155	0.237	0.194	0.223
PR1	0.514	0.435	0.387	0.333	0.358
PR2	0.545	0.440	0.346	0.289	0.244
PR3	0.498	0.456	0.387	0.338	0.277
PR4	0.416	0.385	0.391	0.296	0.330
PR6	0.340	0.232	0.340	0.203	0.254
MTL1	0.360	0.365	0.267	0.238	0.215
MTL5	0.325	0.204	0.326	0.204	0.161
MTL7	0.335	0.254	0.283	0.261	0.205
MTL9	0.398	0.273	0.284	0.221	0.194
MTL10	0.408	0.377	0.276	0.192	0.196
IL2	0.308	0.241	0.316	0.182	0.173
IL3	0.309	0.253	0.281	0.220	0.236
IL6	0.252	0.187	0.283	0.215	0.190
IL7	0.207	0.190	0.244	0.155	0.176
IL10	0.294	0.221	0.255	0.190	0.206
LAE1	0.219	0.184	0.217	0.197	0.272
LAE3	0.139	0.110	0.224	0.174	0.217
LAE4	0.220	0.186	0.238	0.210	0.284
LAE5	0.224	0.189	0.283	0.228	0.250
LAE2	0.252	0.179	0.239	0.172	0.182

Correlations

	EF1	EF3	EF4	EF5	EF6
EF1	1.000				
EF3	0.231	1.000			

EF4	0.359	0.265	1.000		
EF5	0.424	0.179	0.596	1.000	
EF6	0.338	0.241	0.307	0.386	1.000
EW1	0.307	0.160	0.233	0.264	0.298
EW4	0.256	0.038	0.159	0.214	0.185
EW6	0.215	0.156	0.150	0.186	0.200
EW3	0.060	0.171	0.056	0.069	0.129
EW8	0.260	0.102	0.163	0.240	0.185
PR1	0.413	0.110	0.261	0.358	0.249
PR2	0.372	0.047	0.286	0.369	0.180
PR3	0.370	0.098	0.281	0.346	0.206
PR4	0.405	0.163	0.268	0.359	0.251
PR6	0.332	0.186	0.144	0.225	0.143
MTL1	0.340	0.021	0.271	0.303	0.165
MTL5	0.463	0.101	0.269	0.302	0.199
MTL7	0.354	0.174	0.326	0.344	0.230
MTL9	0.337	0.060	0.248	0.311	0.186
MTL10	0.287	-0.031	0.217	0.288	0.155
IL2	0.275	0.034	0.147	0.211	0.106
IL3	0.251	0.100	0.191	0.228	0.136
IL6	0.211	0.090	0.233	0.211	0.087
IL7	0.172	0.014	0.163	0.147	0.021
IL10	0.176	0.077	0.164	0.148	0.079
LAE1	0.161	0.085	0.114	0.116	0.142
LAE3	0.153	0.080	0.167	0.149	0.101
LAE4	0.163	0.034	0.169	0.187	0.141
LAE5	0.259	0.142	0.175	0.182	0.154
LAE2	0.206	0.040	0.085	0.139	0.076

Correlations

	EW1	EW4	EW6	EW3	EW8
EW1	1.000				
EW4	0.433	1.000			
EW6	0.519	0.243	1.000		
EW3	0.298	0.042	0.502	1.000	
EW8	0.466	0.459	0.300	0.217	1.000
PR1	0.244	0.204	0.168	0.045	0.216
PR2	0.185	0.198	0.110	0.003	0.207
PR3	0.262	0.203	0.217	0.093	0.265
PR4	0.273	0.205	0.173	0.118	0.289
PR6	0.167	0.136	0.161	0.062	0.184
MTL1	0.252	0.251	0.146	-0.026	0.279
MTL5	0.202	0.214	0.114	-0.035	0.208
MTL7	0.209	0.152	0.135	0.041	0.134
MTL9	0.222	0.227	0.144	-0.013	0.243
MTL10	0.178	0.220	0.233	0.051	0.171
IL2	0.209	0.230	0.235	0.098	0.202
IL3	0.254	0.243	0.226	0.104	0.214
IL6	0.253	0.200	0.246	0.102	0.239
IL7	0.126	0.170	0.222	0.119	0.103
IL10	0.164	0.184	0.230	0.130	0.119
LAE1	0.346	0.276	0.323	0.188	0.248
LAE3	0.297	0.248	0.236	0.120	0.190
LAE4	0.376	0.355	0.307	0.086	0.301
LAE5	0.319	0.282	0.245	0.090	0.246
LAE2	0.218	0.184	0.331	0.177	0.172

Correlations

PR1	PR2	PR3	PR4	PR6

PR1	1.000				
PR2	0.610	1.000			
PR3	0.560	0.622	1.000		
PR4	0.610	0.553	0.594	1.000	
PR6	0.361	0.336	0.356	0.348	1.000
MTL1	0.319	0.348	0.368	0.279	0.287
MTL5	0.329	0.318	0.325	0.309	0.306
MTL7	0.318	0.274	0.324	0.264	0.271
MTL9	0.352	0.317	0.361	0.301	0.322
MTL10	0.320	0.334	0.311	0.257	0.295
IL2	0.244	0.222	0.301	0.281	0.378
IL3	0.232	0.224	0.244	0.226	0.331
IL6	0.200	0.157	0.221	0.211	0.347
IL7	0.200	0.149	0.156	0.189	0.350
IL10	0.241	0.182	0.211	0.148	0.312
LAE1	0.225	0.131	0.191	0.167	0.239
LAE3	0.154	0.149	0.204	0.119	0.164
LAE4	0.220	0.192	0.223	0.171	0.151
LAE5	0.243	0.161	0.216	0.230	0.243
LAE2	0.225	0.155	0.200	0.190	0.272

Correlations

	MTL1	MTL5	MTL7	MTL9	MTL10
MTL1	1.000				
MTL5	0.396	1.000			
MTL7	0.365	0.405	1.000		
MTL9	0.371	0.379	0.509	1.000	
MTL10	0.387	0.334	0.360	0.425	1.000

IL2	0.313	0.311	0.287	0.369	0.455
IL3	0.304	0.345	0.380	0.389	0.342
IL6	0.284	0.286	0.330	0.290	0.300
IL7	0.302	0.257	0.225	0.254	0.395
IL10	0.301	0.271	0.338	0.346	0.433
LAE1	0.239	0.188	0.243	0.285	0.235
LAE3	0.166	0.144	0.175	0.241	0.154
LAE4	0.260	0.185	0.242	0.335	0.243
LAE5	0.293	0.234	0.342	0.353	0.261
LAE2	0.213	0.138	0.248	0.252	0.383

Correlations

	IL2	IL3	IL6	IL7	IL10
IL2	1.000				
IL3	0.404	1.000			
IL6	0.409	0.541	1.000		
IL7	0.516	0.422	0.439	1.000	
IL10	0.479	0.460	0.418	0.509	1.000
LAE1	0.295	0.411	0.390	0.286	0.408
LAE3	0.171	0.287	0.251	0.157	0.281
LAE4	0.276	0.346	0.284	0.255	0.363
LAE5	0.324	0.389	0.405	0.316	0.424
LAE2	0.394	0.362	0.349	0.385	0.431

Correlations

	LAE1	LAE3	LAE4	LAE5	LAE2
LAE1	1.000				

LAE3	0.511	1.000			
LAE4	0.628	0.636	1.000		
LAE5	0.576	0.497	0.669	1.000	
LAE2	0.475	0.350	0.366	0.429	1.000
